# Draft Genome Sequence of *Amycolatopsis decaplanina* Strain DSM 44594^T^

**DOI:** 10.1128/genomeA.00138-13

**Published:** 2013-04-04

**Authors:** Navjot Kaur, Shailesh Kumar, Monu Bala, Gajendra Pal Singh Raghava, Shanmugam Mayilraj

**Affiliations:** Microbial Type Culture Collection and Gene Bank (MTCC)a and; Bioinformatics Centre,b CSIR-Institute of Microbial Technology, Chandigarh, India

## Abstract

We report the 8.5-Mb genome sequence of *Amycolatopsis decaplanina* strain DSM 44594^T^, isolated from a soil sample from India. The draft genome of strain DSM 44594^T^ consists of 8,533,276 bp with a 68.6% G+C content, 7,899 protein-coding genes, and 57 RNAs.

## GENOME ANNOUNCEMENT

The genus *Amycolatopsis* was proposed by Lechevalier et al. in 1986 (1). *Amycolatopsis decaplanina* was proposed by Wink et al. in 2004 (2). *A. decaplanina* strain DSM 44594^T^, which was obtained from the open collection of the German Collection of microorganisms and Cell Cultures (DSMZ), Braunschweig, Germany, deposited by Joachim M. Wink, is a Gram-positive, aerobic, nonmotile, catalase-positive actinomycete that forms extensively branched substrate mycelia. The genome of *A. decaplanina* strain DSM 44594^T^ was sequenced using the Illumina-HiSeq 1000 paired-end technology and produced a total of 76,665,954 paired-end reads (insert size of 350 bp) of 101 bp. We used the Next-Generating Sequencing Quality Control (NGS QC) toolkit v2.3 (3) to filter the data for high-quality (HQ) (cutoff read length for HQ = 70%, cutoff quality score = 20) vector- and adaptor-free reads for genome assembly. A total of 70,826,953 high-quality vector-filtered reads (~794× coverage) were used for assembly with SOAP*denovo* v1.05 (at a hash length of 73), followed by GapCloser (at a hash length of 27) software (4). The final assembly contains 85 contigs of total size 8,533,276 bp, with an N_50_ contig length of 302.8 kb; the largest contigs assembled measure 791.1 kb.

The draft genome (85 contigs) comprising 8,533,276 bp was annotated with the help of the Rapid Annotations using Subsystems Technology (RAST) server (5). A total of 7,899 predicted coding regions (CDSs), 3 rRNAs, and 54 tRNAs were predicted. RAST annotation indicates that the strains *Streptomyces* sp. AA4 (score 530), *Actinosynnema mirum* DSM 43827 (score 448), *Saccharomonospora viridis* DSM 43017 (score 419), and *Saccharopolyspora erythraea* NRRL 2338 (score 415) are the closest neighbors of strain DSM 44594^T^. Annotation available at the RAST server also indicates that the strain DSM 44594^T^ has the genes coding for glycolysis and gluconeogenesis, the Entner-Doudoroff pathway, and the tricarboxylic acid (TCA) cycle. The genes coding for alkaline phosphatase, galactosidase (alpha and beta subunits), mannosidase (alpha and beta subunits), urease (alpha, beta, and gamma), catalase (EC 1.11.1.6), and ferroxidase (EC 1.16.3.1) are also present in the genome annotation of strain DSM 44594^T^. We have mapped all predicted 7,899 CDSs to the Kyoto Encyclopedia of Genes and Genomes (KEGG) pathways (6) with the help of KEGG Automatic Annotation Server (KAAS) (7) and found the genes coding for glucose-1-phosphate thymidylyltransferase (EC 2.7.7.24), dTDP-glucose 4,6-dehydratase (EC 4.2.1.46), and dTDP-4-dehydrorhamnose 3,5-epimerase (EC 5.1.3.13) mapped on the pathway of polyketide sugar unit biosynthesis. The genes coding for salicylate biosynthesis isochorismate synthase (EC 5.4.4.2), isochorismate pyruvate lyase (EC 4.2.99.21), bifunctional isochorismate lyase/aryl carrier protein (EC 3.3.2.1), 2,3-dihydro-2,3-dihydroxybenzoate dehydrogenase (EC 1.3.1.28), 2,3-dihydroxybenzoate-AMP ligase (EC 2.7.7.58), and pyochelin synthetase mapped on the pathway of the biosynthesis of siderophore group nonribosomal peptides.

### Nucleotide sequence accession numbers. 

The draft genome sequence of *A. decaplanina* strain DSM 44594^T^ has been included in the GenBank Whole-Genome Shotgun (WGS) database under the accession no. AOHO00000000. The version described in this paper is the first version, accession no. AOHO01000000.
